# Metal Complexes of Omadine (*N*-Hydroxypyridine-2-thione): Differences of Antioxidant and Pro-Oxidant Behavior in Light and Dark Conditions with Possible Toxicity Implications

**DOI:** 10.3390/molecules28104210

**Published:** 2023-05-20

**Authors:** Olga Yu. Selyutina, Viktor A. Timoshnikov, Nikolay E. Polyakov, George J. Kontoghiorghes

**Affiliations:** 1 Institute of Chemical Kinetics & Combustion, 630090 Novosibirsk, Russia; olga.gluschenko@gmail.com (O.Y.S.); timoshnikov@kinetics.nsc.ru (V.A.T.); polyakov@kinetics.nsc.ru (N.E.P.); 2 Institute of Solid Chemistry and Mechanochemistry, 630090 Novosibirsk, Russia; 3 Postgraduate Research Institute of Science, Technology, Environment and Medicine, CY-3021 Limassol, Cyprus

**Keywords:** omadine, chelating drug, chelator, antioxidant activity, ROS, pro-oxidant activity, lipid oxidation, photoactivity, toxicity

## Abstract

Omadine or *N*-hydroxypyridine-2-thione and its metal complexes are widely used in medicine and show bactericidal, fungicidal, anticancer, and photochemical activity. The redox activity of omadine complexes with iron, copper, and zinc on lipid peroxidation under light and dark conditions has been investigated. The monitoring of the oxidation of linoleic acid micelles, resembling a model of lipid membrane, was carried out using nuclear magnetic resonance (^1^H-NMR). It has been shown that the omadine–zinc complex can induce the oxidation of linoleic acid under light irradiation, whereas the complexes with iron and copper are photochemically stable. All the chelating complexes of omadine appear to be redox-inactive in the presence of hydrogen peroxide under dark conditions. These findings suggest that omadine can demonstrate antioxidant behavior in processes involving reactive oxygen species generation induced by transition metals (Fenton and photo-Fenton reactions). However, the omadine complex with zinc, which is widely used in shampoos and ointments, is photochemically active and may cause oxidative cell membrane damage when exposed to light, with possible implications to health.

## 1. Introduction

Iron, copper, and zinc are essential metals that play vital roles in living organisms, including humans. Metalloproteins containing these metals perform a variety of functions in cells. In particular, iron participates in oxygen transport and in energy transduction [1,2,3,4], while copper plays a vital role in enzymatic and catalytic functions [5,6]. Under normal conditions, iron and copper catalytic centers participate in metabolic pathways involving free radical formation, which is controlled by homeostatic mechanisms including antioxidant enzymes and molecules [1,2]. However, the presence of excess iron or copper in the case of various pathological conditions such as thalassemia, sickle cell anemia, idiopathic hemochromatosis, and Wilson’s disease leads to serious toxic side effects and fatalities [2]. Under these conditions, excess iron and copper ions participate in redox reactions, with the uncontrolled formation of reactive oxygen species (ROS), which cannot be balanced by antioxidant mechanisms or molecules [2,7,8,9]. Reactive oxygen species are able to react with lipids, DNA, sugars, proteins, and other molecules, causing their oxidation and irreversible damage [10,11].

One of the types of cell death induced by iron is ferroptosis [12,13,14,15]. The mechanism of this process is associated among other changes with the oxidation of lipids in the cell membrane as a result of general redox imbalance caused by iron catalysis [12,13,14,15]. Ferroptosis is a programmed cell death process, which has been associated with various diseases such as cancer and immune system, neurodegenerative, heart, and other diseases [16,17,18,19]. In all these different diseases, ferroptotic programmed cell death is induced by iron in normal healthy cells and involves oxidative stress. Effective iron chelators could inhibit ferroptosis including redox reactions with iron ions [12,19,20,21,22]. However, selected lipophilic chelators and their iron complexes have been proposed to be used as anticancer therapeutics by inducing ferroptosis in drug-resistant tumors [22].

In contrast to the “free” metal ion, the reactivity and metabolic pathways for the complexed metal ion can differ significantly [19,22,23]. In this context, the antioxidant/pro-oxidant effects of the iron-selective chelating drugs deferiprone, deferoxamine, and deferasirox have been previously studied under different conditions [24,25,26,27]. These studies were aimed at finding an effective drug for inhibiting oxidative stress toxicity, including related mechanisms associated to ferroptosis. However, there is no sufficient information in the literature about studies regarding the effect of chelating drugs and other chelators on different aspects of ferroptosis. Such information may have both fundamental and practical implications, especially for the treatment of cancer and other diseases [19,22].

**Figure 1:**
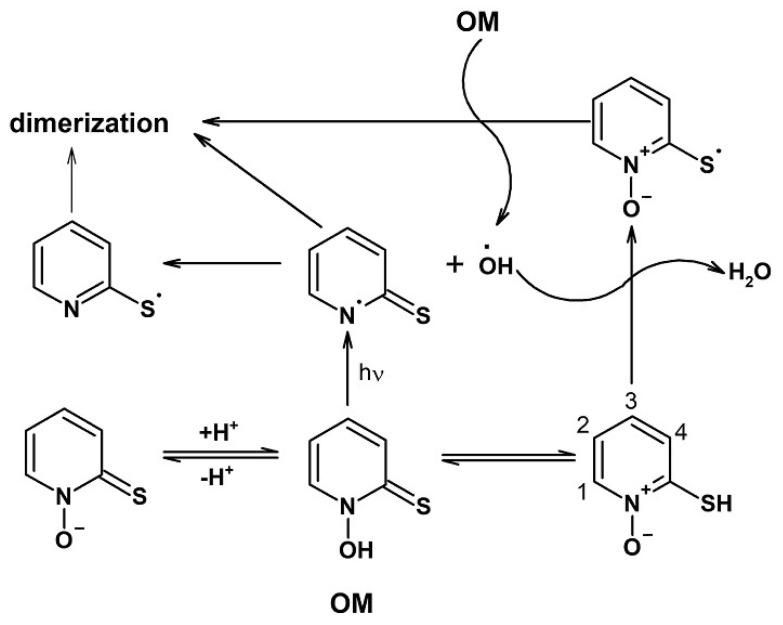
Schematic representation of the photochemical decomposition of omadine (OM). Modification of the OM molecule is shown, following exposure to light and formation of photo-degradation products. The numbers denote the position of protons in the OM molecule. The scheme is based on information provided in the references [37,38,39,40].

Omadine (*N*-hydroxypyridine-2-thione, OM) (Figure 1) is a biologically active chelating drug of the pyrithione group [22,23,28,29]. Both the chelator and its complexes with metal ions, have been shown to have anticancer, fungicidal and bactericidal activities [29,30,31,32,33,34,35,36]. Moreover, OM is also a photochemically active substance. Under irradiation, OM is decomposed, causing the formation of sulfur-centered radicals and also hydroxyl radicals (·OH). In further reaction steps, sulfur-centered radicals are able to recombine with the formation of different products or participate in radical reactions with other radicals and molecules. In addition, the highly reactive hydroxyl radicals can react with almost all biomolecules and also with OM itself, causing their oxidation (Figure 1) [37,38,39,40]. For example, the effectiveness of OM as an oxidant in photo-Fenton reactions has been shown in experimental models using DNA and carotenoids [38,41].

It should be noted that among the OM chelate complexes, the most studied is the omadine–zinc (OM–Zn) complex, which is widely used as the active ingredient in medicinal ointments and shampoos for the treatment of fungal and bacterial infections [42,43,44]. Different studies have shown that zinc enhances the membranotropic activity of OM, allowing an increase in OM–Zn penetration into the cells. Subsequently, OM is able to bind to different metals, for example, to the copper and iron present in cells due to transmetallation, leading to the possible participation of chelate complexes in intracellular activities including redox reactions, the inhibition of DNA, and also protein activity [45]. Similar transmetallation effects in the use of zinc have also been described in studies with other chelators such as the thiosemicarbazones [46,47]. Furthermore, it appears that zinc could demonstrate antioxidant activity due to competitive binding with various sulfur-containing metalloproteins, thereby inhibiting oxidative stress [48,49,50]. Another antioxidant property proposed for zinc is the stabilization of the cell membrane, but the exact mechanism has not yet been fully defined [49].

The main goal of this work is to study the antioxidant/pro-oxidant activity of OM and its complexes with iron, copper, and zinc ions in photo-Fenton and lipid peroxidation reactions using the nuclear magnetic resonance (^1^H-NMR) technique. In this context, linoleic acid (LA) micelles were selected as a model resembling a lipid membrane.

## 2. Results

### 2.1. Photo-Induced Oxidation of Linoleic Acid Micelles in the Presence of Omadine and Its Metal Complexes

The absorption spectra and extinction coefficients (ε) of OM, OM–Zn, OM–Cu, and OM–Fe at a laser wavelength of 308 nm have been previously measured and reported as follows: ε_308nm_(OM) = 1150 sm^−1^M^−1^; ε_308nm_(OM–Zn) = 2200 sm^−1^M^−1^; ε_308nm_(OM–Cu) = 5250 sm^−1^M^−1^; ε_308nm_(OM–Fe) = 1530 sm^−1^M^−1^ [51,52]. In the present study, the photochemical activity of OM and its metal complexes in the photo-oxidation of lipid membranes was studied using LA micelles. Lipid micelles including those of LA were widely used in previous studies as a model for mimicking the properties of biological membranes [53]. Similarly, the peroxidation of LA, which is a polyunsaturated fatty acid, has also been previously described [53,54].

**Figure 2:**
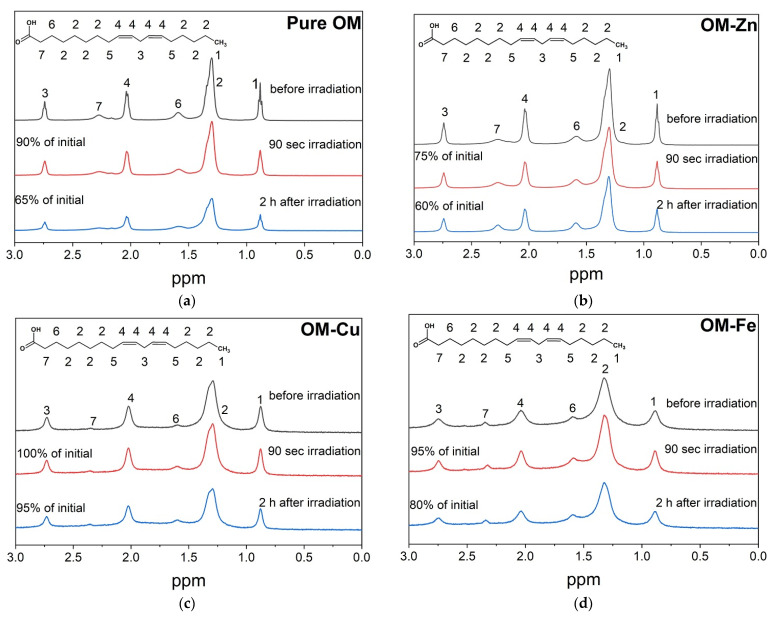
^1^H-NMR spectra of LA (7 mM) + OM (1 mM) (**a**); + ZnCl_2_ (0.5 mM) (**b**); + CuCl_2_ (0.5 mM) (**c**); + FeSO_4_ (0.5 mM) (**d**) in D_2_O (pH = 6) before and after irradiation (308 nm). The peaks in the spectra correspond to the LA protons. Each sample was irradiated for 90 s (90 laser pulses). All experiments were carried out at a temperature of 303 K.

The photo-induced oxidation of LA in the presence of OM and its metal complexes was investigated in the present study using ^1^H-NMR spectroscopy. Figure 2 shows the ^1^H-NMR spectra of LA micelles in the presence of OM and its complexes with Zn^2+^, Cu^2+^ and Fe^2+^. No decrease in the intensity of the LA signals was observed in the absence of OM. On the other hand, a significant decrease in the intensity of LA signals in the presence of OM was observed after irradiation (Figure 2a). It appears that the decrease in the signal intensity in the presence of OM may have been caused following the reaction with hydroxyl radicals, which are formed during the OM photo-degradation (Figure 1) [37,38,39,40]. In the absence of metal ions, a decrease in the intensity of about 10% was observed during the irradiation time (90 s). Following irradiation, the samples were kept in dark conditions and the total decrease in signal intensity over a two-hour period was estimated as 35%. The addition of Zn^2+^ enhanced the decrease in the signal intensity during irradiation up to 25%, but the total decrease during the two-hour period was close to that of OM without metal ion incubation (40%). In contrast, addition of the Fe^2+^ and Cu^2+^ decreased the pro-oxidant photo-activity of OM. The total decrease in signal intensity in the presence of Cu^2+^ and Fe^2+^ was estimated as 5% and 20%, respectively.

**Figure 3:**
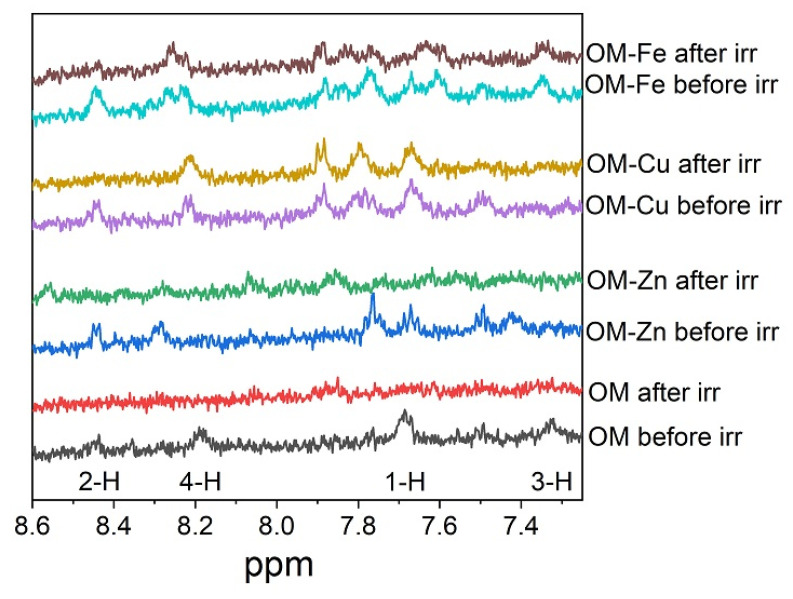
Fragments of ^1^H-NMR spectra of OM (1mM) in LA (7 mM) micelles in D_2_O (pH = 6) in the absence and in the presence of ZnCl_2_, CuCl_2_ or FeSO_4_ (0.5 mM) before and after 90 s of irradiation (irr) (308 nm, 90 laser pulses). The signals in the spectrum correspond to the neutral zwitterion form of OM as shown in Figure 1. All experiments were carried out at a temperature of 303 K.

Figure 3 shows fragments of ^1^H-NMR spectra containing the signals of OM in LA micelles. In the absence of metal ions, four signals corresponding to four OM protons are observed. In the presence of metal ions, the number of signals increases, indicating the presence of complexes with various stoichiometry and/or complexes with several forms of OM (neutral, neutral zwitterion, anion) in the samples (Figure 1). Unfortunately, the accurate identification of additional signals for OM complexes was difficult due to the significant broadening of the NMR lines.

It is noteworthy that in the absence of metal ions and in the presence of only zinc ions, the OM signals totally disappeared from the spectrum after irradiation, which indicates the complete decomposition of OM. In the case of iron and copper complexes, only part of the signals disappears from the spectrum, indicating that some form of OM complexes with iron and copper exhibits higher photo-stability than pure OM, which apparently leads to the inhibition of the photo-induced oxidation of LA.

**Figure 4:**
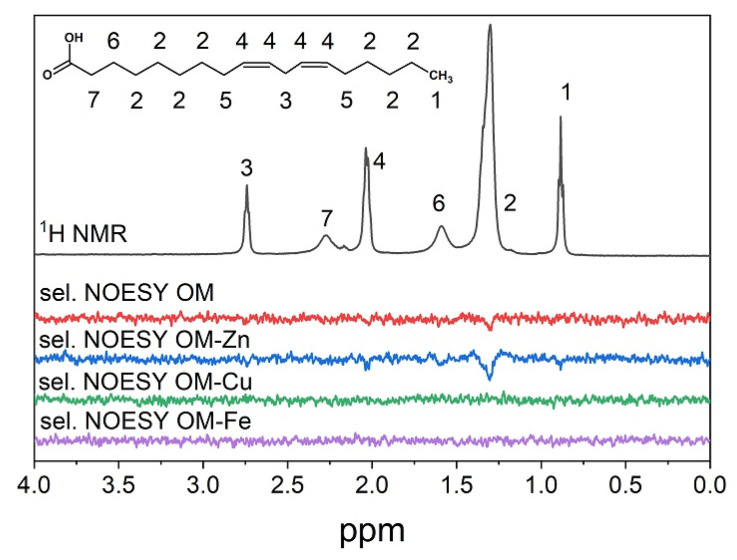
Fragments of the 1D Nuclear Overhauser Effect Spectroscopy (NOESY) and NMR spectra (black line) of OM (1 mM) and its complexes with ZnCl_2_, CuCl_2_ or FeSO_4_ (0.5 mM) in LA micelles (7 mM) at pH = 6 and at a temperature of 303 K. Selective excitations of all OM protons was performed.

Additionally, selective NOESY experiments were carried out to determine the localization of OM and its complexes in the LA micelles (Figure 4). Cross-peaks in the NOESY spectrum are observed between the nuclei located at a distance of less than 0.4 nm. For example, in the case of OM, cross-peaks between OM protons and CH_2_-groups of LA were observed. In the case of zinc complexes, additional cross-peaks between OM protons and proton 6 of LA near double bonds and terminal CH_3_-groups were observed. However, no cross-peaks with lipids signals were observed in the case of copper and iron complexes. Overall, it can be suggested from these observations that OM is located inside the LA micelles, its complexes with zinc penetrate deeper into micelles, and its complexes with copper and iron do not penetrate inside micelles. This may be another reason for the lower photo-activity of OM complexes with copper and iron.

### 2.2. Peroxidation of Linoleic Acid Micelles in the Presence of Omadine and Its Metal Complexes in Dark Conditions

**Figure 5:**
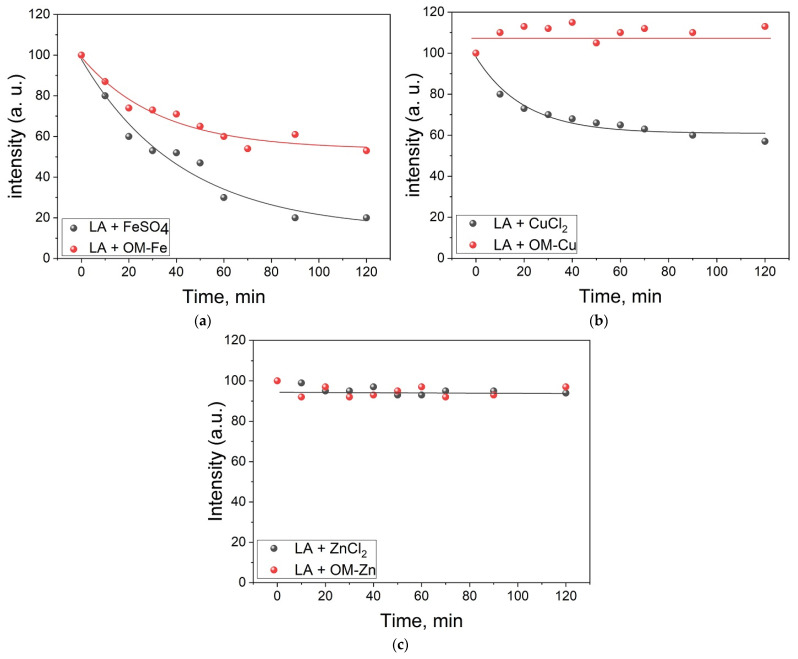
Kinetic profile of linoleic acid peroxidation in the presence of omadine complexes with iron, copper and zinc ions. The experimental conditions were as follows: 7 mM LA + 1 mM OM + 0.5 M H_2_O_2_, plus (**a**) FeSO_4_ (0.5 mM), (**b**) CuCl_2_ (0.5 mM), (**c**) ZnCl_2_ (0.5 mM), all in D_2_O at pH = 6. All experiments were carried out at a temperature of 303 K. The graphs were plotted using the decay of the integral intensity signal of the LA protons (proton 3) at 2.7 ppm (a.u.—arbitrary units).

Further studies were carried out to examine the redox activity of OM complexes with metal ions in the peroxidation reactions of LA, which in the present study was used as a general model of lipid peroxidation [53,54] (Equations (1)–(8)). The effectiveness of the OM complexes in this reaction was monitored using ^1^H-NMR spectra at different time intervals. Furthermore, this procedure allowed for the measurement of the kinetic changes in the integral intensity of the LA protons. The same approach was previously described in more detail [27]. In this context, the time dependence of the intensity of the NMR signal was measured by monitoring the changes of the bis-allylic protons of LA (2.7 ppm, signal 3 in Figure 4). Since the initiation stage of LA oxidation involves the abstraction of a hydrogen atom at this position, whereas the reaction products (lipid radicals and conjugated dienes, Equations (1)–(8)) do not contain such protons in the structure, it can be suggested that the initiation stage leads to a decrease in the intensity of this signal as a function of time (Figure 5).

The schematic representation of iron- and copper-induced lipid peroxidation is described in Equations (1)–(8) below. Similar mechanisms are suggested for both the iron- and copper-induced Fenton reaction. The peroxidation of lipids (LH) involves a number of reactions in the presence of hydrogen peroxide [55,56,57,58].

(1) $${Fe}^{2+}+H_{2}O_{2}\to {Fe}^{3+}+\dot{OH}+{OH}^{-}$$

(2) $${Fe}^{3+}+H_{2}O_{2}\to {Fe}^{2+}+\dot{OOH}+H^{+}$$

(3) $${Cu}^{+}+H_{2}O_{2}\to {Cu}^{2+}+\dot{OH}+{OH}^{-}$$

(4) $${Cu}^{2+}+H_{2}O_{2}\to {Cu}^{+}+\dot{OOH}+H^{+}$$

(5) $$LH+\dot{OH}\to \dot{L}+H_{2}O$$

(6) $$\dot{L}+O_{2}\to \dot{LOO}$$

(7) $$\dot{LOO}+LH\to LOOH+\dot{L}$$

(8) $$LOOH\to \dot{LO}\to epoxides,hydroperoxides,aldehydes$$

The experimental points in Figure 5 were approximated to fit an exponential decay, and subsequently the reaction rate constants of LA peroxidation were calculated from the fitting parameters. In this context, the rate constant of the initiation stage of the LA peroxidation in the presence of FeSO_4_ was estimated to be 5.2 ± 0.2 × 10^−4^ s^−1^. In the presence of OM–Fe, the rate constant decreased to 4.0 ± 0.2 × 10^−4^ s^−1^. In the case of copper, the rate constant in the absence of OM was estimated as 4.5 ± 0.2 × 10^−4^ s^−1^, whereas in the presence of OM–Cu the reaction was completely inhibited. However, in the case of zinc, which is a non-transition metal, no changes related to LA signal intensity were observed both in the absence and presence of OM.

Finally, in the control experiments using the mixture of LA plus ZnCl_2_ or FeSO_4_ or CuCl_2_ in the absence of H_2_O_2_, there was no decrease in the intensity of LA signals in the spectrum over 24 h at a temperature of 303 K.

## 3. Materials and Methods

### 3.1. Materials

Omadine (OM, 99%), ferrous sulphate (FeSO_4_·6H_2_O, 99%), copper chloride (CuCl_2_·2H_2_O, 99%), zinc chloride (ZnCl_2_·2H_2_O, 99%), and H_2_O_2_ (35.5%) were obtained from Sigma-Aldrich (St. Louis, MO, USA). Linoleic acid (LA, purity > 99.0%) was purchased from Shanghai Aladdin Bio-Chem Technology Co., Ltd., (Shanghai, China). All compounds were used as received. Deuterated solvent (D_2_O, 99.8% D) was obtained from Solvex-D Co. (Moscow, Russia) and was used as supplied. All experiments were carried out at a temperature of 303 K.

### 3.2. Methods

#### 3.2.1. The ^1^H-NMR Study of Lipid Peroxidation

The study of lipid peroxidation was carried out using a reaction mixture that consisted of LA micelles (7 mM), H_2_O_2_ (0.5 M), OM (1 mM), and FeSO_4_, ZnCl_2,_ or CuCl_2_ (0.5 mM) in deuterated water (pH 6). The ^1^H-NMR spectra were recorded using a Bruker Avance HD III NMR spectrometer (500 MHz) (Rheinstetten, Germany). Omadine was mixed with LA in chloroform, then the solvent was evaporated, and the remaining film was hydrated in deuterated water of pH 6 (pKa of OM is 4.6). Then, one of the salts (FeSO_4_, ZnCl_2_ or CuCl_2_) was added and the sample was incubated for 30 min to establish equilibrium. The reaction was initiated by the addition of H_2_O_2_ into the solution mixture. This method has been described in more detail in a previous study [59].

#### 3.2.2. The ^1^H-NMR Study of the Photo-Oxidation of Linoleic Acid

The study of LA photo-oxidation was carried out using a reaction mixture that consisted of LA micelles (7 mM), OM (1 mM) and FeSO_4_, ZnCl_2_ or CuCl_2_ (0.5 mM) in deuterated water (pH 6). The preparation method of LA micelles is the same as that described in the previous section. An EMG 101 MSC Lambda Physik excimer laser was used as the light source for photo-oxidation experiments (λ = 308 nm, pulse duration 15 ns, average pulse energy100 mJ). The pulse frequency used was 1 Hz.

#### 3.2.3. The ^1^H-NMR Study of the Penetration of Omadine and Its Metal Complexes into Micelles

The ^1^H-NMR and the selective Nuclear Overhauser Effect Spectroscopy (NOESY) methods were used to examine the penetration of OM and its chelate complexes with iron (OM–Fe), copper (OM–Cu), and zinc (OM–Zn) ions into LA micelles. The NMR spectra were recorded on Bruker Avance HD III NMR spectrometer (500 MHz ^1^H operating frequency). T_1_ relaxation times were measured using a standard inversion-recovery pulse sequence. All experiments were carried out at a temperature of 303 K.

## 4. Discussion

There are many different biological and toxicity targets, which can be affected by the action of various drugs possessing pro-oxidant or antioxidant properties. Some of these drugs, such as doxorubicin, are commonly used to treat cancer and also other conditions [19,22,60,61,62]. One of the targets for designing anticancer drugs is DNA, which is a vital biomolecule for cell viability and functioning [62,63,64,65]. Nevertheless, the cell membrane is also considered as a potential target for oxidative activity in cancer treatment [66,67,68,69]. Recently, the ferroptotic mechanism of programmed cell death, which involves the generation of ROS and the oxidative destruction of cell membranes by iron, has also been a major target under investigation [17,70,71]. However, there is no sufficient information thus far on the initiation and/or enhancement of ferroptosis caused by drugs that have chelating metal capability and pro-oxidant activity.

Omadine was proposed as a potential drug capable of initiating and accelerating the process of oxidative stress toxicity, including ferroptotic cell death. Several properties of OM fulfill this role, including the binding of metal ions, such as iron, copper, and zinc; membranotropic activity; and also the pro-oxidant activity in photochemical reactions with the formation of hydroxyl radicals [29,72,73,74,75]. There are many studies reported regarding the possible use of OM and its metal complexes as potential anticancer drugs [2,30,31,32,33,44,45,73,75]. However, the mechanisms of the redox activity of the OM metal complexes have not been previously described. In addition, despite the reports of many studies on the photochemical activity of OM, the photochemical activity of OM metal complexes has not yet been fully investigated. In this context, the study of the redox activity of OM and its metal complexes in dark and photochemical reactions involving lipids may have important pharmacological and toxicological implications, especially in relation to the influence of the OM metal complexes on bio-molecular mechanisms, including ferroptosis.

A comparative analysis of OM and its metal complexes in the photo-oxidation activity of LA micelles has shown low efficiency for OM–Fe and OM–Cu complexes compared to OM–Zn complexes, as well as compared to OM itself (Figure 2). It is known that many chelate complexes with iron, including aqua complexes, are photoactive and can participate in photo-induced reactions with the formation of reactive oxygen radicals [76,77,78]. It was somewhat unusual to see the display of antioxidant activity by OM in the presence of iron ions. This observation can be explained by the low membranotropism of OM–Fe and OM–Cu complexes, which was proved by the selective NOESY method in the present study (Figure 4). Previous studies have suggested that the mechanism of penetration of the OM–Fe and OM–Cu chelate complexes into cells is based on the participation of membrane-transport metal-binding proteins [72].

Similarly, it was also previously suggested that the OM–Zn complexes are able to interact with lipids inside membranes, disrupting their organization and uncoupling them [79]. The results of the present study, which were obtained using the NOESY method, suggest that the OM–Zn complex demonstrates the highest penetration ability into the LA micelles among all other OM metal complexes.

An additional feature of the OM metal complexes with iron and copper ions is their higher photostability. As mentioned above, OM under UV irradiation can be decomposed with the formation of hydroxyl radicals [37,38,39,40]. It appears that upon the irradiation of OM and the OM–Zn complex in LA micelles, the OM NMR signals disappear from the spectra and the integral intensity of LA protons decreases (Figure 3), which supports the mechanism that the generated hydroxyl radicals participate in the redox reactions of LA oxidation (see Equations (1)–(8)). Even after irradiation, the integrated signal intensity continues to decrease due to the chain reaction of the formation of various dimers and other LA oxidation products (see Equations (1)–(8) and Figure 2a,b) [55,56,57]. It can be suggested that the higher photostability of the OM complexes with iron and copper ions can be due to their possible protonation, thereby hindering the passage of the complex into the membrane.

The peroxidation of LA micelles in the presence of the OM complexes with iron, copper, and zinc ions has also been studied under dark conditions using ^1^H-NMR.

As is shown in Figure 5, all OM complexes exhibit antioxidant activity under such conditions. It was also observed that the rate of decay of the integral intensity of LA decreases significantly when the chelator is added to the solution. The overall decrease in the redox activity of iron and copper ions during complex formation with OM can be explained by the low membrane permeability of these chelate complexes into LA micelles. This suggestion is supported by the results of the selective NOESY study (see Figure 4). However, it has been previously shown that the cell membrane permeability of OM–Fe is much higher in cell studies, suggesting a different mechanism of membrane transport [72,80,81]. In the case of OM–Zn, the oxidation of LA has not taken place because zinc is a non-transition metal and does not participate in the Fenton reaction. It is also important to clarify that OM itself did not degrade, indicating a lower rate constant of OM oxidation by hydroxyl radicals in comparison to the rate constant of LA oxidation.

Lipid peroxidation is widely studied using various other techniques in addition to ^1^H-NMR, such as UV-Vis spectroscopy and Raman spectroscopy [82,83,84]. In each case, the use of such techniques could provide important information for monitoring different aspects of the oxidation of lipids [82,83,84]. For example, UV-Vis spectroscopy could be used to detect malondialdehyde accumulation, which is one of the terminal products of peroxidation reactions, whereas the ^1^H-NMR method used in this study provides information on the initial stages of the reaction.

**Figure 6:**
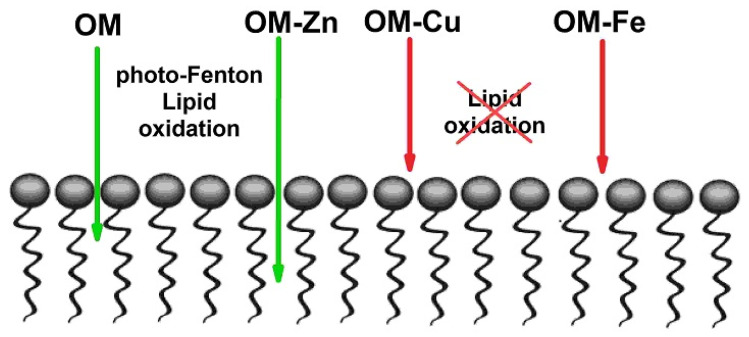
Cardoon illustration showing different abilities between omadine (OM) and its metal complexes (OM–Zn, OM–Cu and OM–Fe), in the penetration of linoleic acid (LA) micelles and the interaction with lipids.

Overall, it can be suggested that the main cause of the low redox activity of the OM metal complexes with copper and iron is their low LA micelle membranotropic ability. In such cases, ligands and complexed metals cannot penetrate close enough to the LA biotargets of ROS attack to initiate and maintain the cascade of redox reactions (Figure 6).

## 5. Conclusions

Molecular studies including the redox effects of OM and its metal complexes with iron, copper, and zinc are of major importance for determining the general pharmacological and toxicological properties of OM, as well as all other chelating drugs. Furthermore, the findings from such studies may have therapeutic or toxicological implications for people receiving OM-containing pharmaceutical and cosmetic products.

It has been shown in this study that OM cannot enhance the pro-oxidant properties of iron and copper ions, but instead can act as an antioxidant in this experimental LA micelle membrane model. In general, the binding of iron and copper ions by OM prevents the transfer of these metal–OM complexes inside LA micelles and their participation in dark and photo-induced redox reactions. In contrast, only OM and OM–Zn complexes show pro-oxidant activity in the photo-oxidation reaction of LA micelles.

In general, OM shows much lower efficiency for antioxidant potential in comparison to other chelating drugs of iron and copper ions, such as deferiprone, in both dark and photo-induced reactions using the same model system.

The mechanism of anticancer activity of OM–iron complexes, which has been shown in many cancer cell lines, has not been elucidated in the present study. However, it appears that the anticancer mechanism is not associated with free radical activity and processes involving the lipid peroxidation of the cell membrane.

Nevertheless, this study is of great fundamental and applied importance for understanding the mechanism of the influence of various chelators in ferroptosis and other similar processes.

The finding that the OM complex with zinc ions is photochemically active and may cause oxidative cell membrane damage when exposed to light is of major toxicological importance. Considering that the OM–Zn complex is widely used in shampoos and ointments, it can be suggested that such products may have adverse implications on health, unless such medications are stored in the dark.

## Data Availability

Data is contained within the article.

## Abbreviations

NOESY	Nuclear Overhauser Effect Spectroscopy
NMR	Nuclear magnetic Resonance
OM	Omadine
LA	Linoleic Acid
ROS	Reactive Oxygen Species
